# A Review on Methods for Random Motion Detection and Compensation in Bio-Radar Systems

**DOI:** 10.3390/s19030604

**Published:** 2019-01-31

**Authors:** Carolina Gouveia, José Vieira, Pedro Pinho

**Affiliations:** 1 Instituto de Telecomunicações, 3810-193 Aveiro, Portugal; jnvieira@ua.pt (J.V.); ppinho@deetc.isel.pt (P.P.); 2 Departamento de Eletrónica, Telecomunicações e Informática, Universidade de Aveiro, 3810-193 Aveiro, Portugal; 3 Departamento de Engenharia Eletrónica, Telecomunicações e de Computadores, Instituto Superior de Engenharia de Lisboa, 1959-007 Lisboa, Portugal

**Keywords:** bio-signals, Doppler, radar, random motion, continuous wave radar

## Abstract

The bio-radar system can measure vital signals accurately, by using the Doppler effect principle, which relates the received signal properties to the distance change between the radar antennas and the subject chest-wall. These systems have countless applications, from short range detection to assist in rescue missions, to long-term applications as for the continuous sleeping monitoring. Once the main applications of these systems intend to monitor subjects during long periods of time and under noisy environments, it is impossible to guarantee the patient immobilization, hence its random motion, as well as other clutter sources, will interfere in the acquired signals. Therefore, the signal processing algorithms developed for these applications have been facing several challenges regarding the random motion detection and mitigation. In this paper, an extended review on the already implemented methods is done, considering continuous wave radars. Several sources of random motion are considered, along with different approaches to compensate the distortions caused by them.

## 1. Introduction

The contactless measurement of bio-signals has the potential to improve many areas. In the medical field, among many other applications, it can be highlighted the continuous monitoring of vital signals in bedridden patients, as in the burn units in hospitals, where physical contact with the patient is totally discouraged. Also for sleeping monitoring, namely to support cases of Obstructive Sleep Apnea (OSA) syndrome without interfering with the normal life style of the patients, or in the prevention of Sudden Infant Death Syndrome (SIDS) [1]. In terms of commercial applications, it can be highlighted the vehicular area, where the driver’s vital signals are monitored to avoid any possible accident in case of cardiac failure or these systems usage in ambulances to minimize the direct contact with critical patients. Applications in psychology are also possible, as for example the measurement of stress response [2].

Thus, it is possible to define the *Bio-Radar* system as a technology capable to acquire vital signals, such as the respiratory signal and the cardiac signal, without interfering directly with the patient. For this purpose, it uses electromagnetic waves, which are transmitted towards to the chest-wall of the subject under monitoring, and the reflected echo is received. From the Doppler effect it is possible to relate the received signal properties with the distance change between the radar antennas and the subject chest-wall, which moves according to the cardiopulmonary function.

**Figure 1:**
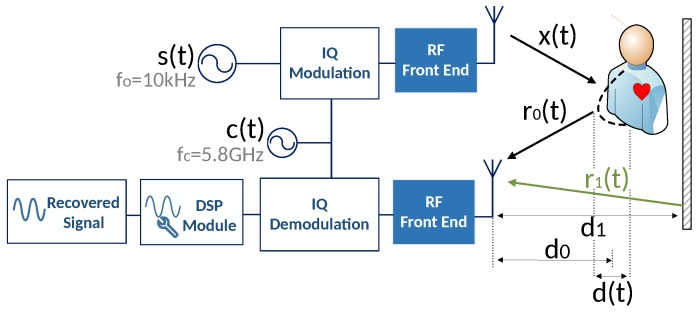
Bio-Radar system block diagram [3].

In order to better understand its operation principal, an example of a bio-radar’s system is represented by the block diagram in Figure 1. This example is composed by a Continuous Wave (CW) Doppler radar which continuously transmits a sinusoidal carrier, generated digitally, and receives the echo from the reflecting target. Due to the Doppler effect, there is a phase change as the subject’s chest-wall moves towards or away from the radar and hence a phase modulation in the received signal is created [1].

The concept of non-contact extraction of human physiological parameters, has been demonstrated by pioneers during the 1970s and its state-of-the-art have been following a time line of hardware implementation. The pioneers in [4,5,6] measured both the respiration and heartbeat separately during apnea interspersed periods. Then, the followed prototypes were implemented with transceivers composed by single Radio Frequency (RF) hardware components interconnected with each other [7]. Thenceforth, these systems were implemented using incorporated analog and digital signal processing and thereafter the RF front-end components were integrated in a single chip using CMOS processes [8]. Some authors have published manuscripts that gather the bio-radar hardware implementation state-of-the-art and they are presented in [1,9].

Presently, the research in this area is even more focused on the development of systems with features that guarantee low power, small dimensions, better accuracy, long range detection and more robust operation. With this in mind, in [10] a bio-radar implementation was proposed using a front-end based on a Software Defined Radio (SDR) system. These radars allow the digital configuration of its input and output (receiver and transmitter), regarding the required frequency and sampling rate of the current application. These configurable systems present an advantage compared to the previously referred ones in [4,5,6,7,8], due to its flexibility and compact features. They are also based on homodyne receivers, which are capable to use the same source in both transmitter and receiver and thus guarantee the range correlation and avoid the phase noise [11].

In [10], signals were first acquired, recorded in binary files and then processed offline using MATLAB. An improve of that prototype, was proposed in [12], where it could be possible to operate in real time, using the LabVIEW software for Digital Signal Processing (DSP) algorithm execution. In this way, the acquired signals could be processed and visualized during their acquisition time. The developed prototype was validated in [3], using a certified measurement equipment, named BioPac MP100. The signals were acquired using the bio-radar prototype and the BioPac MP100 simultaneously and compared afterwards. Both acquisitions revealed approximated waveforms and breathing rates. Moreover, in [13] the influence of the antennas design on the extracted signal was verified. For this purpose, directive and non-directive antennas were used to acquire signals and it was concluded that the system is more sensitive to clutter if non-directive antennas are used. Thus, assuming that the desired target is within the radar range, the usage of directive antennas is recommended, so the transmitted power is more focused at the subject’s chest-wall and less parasitic reflections are received. However, it is important to note that the usage of narrow beam width hampers the alignment with the optimal point of detection (the chest-wall center in the respiratory signal case), hence other techniques should be implemented to steer the antenna’s beam in the right direction.

Bio-Radar systems have been implemented using different radar operation modes, beside the CW such as Frequency-Modulated Continuous Wave (FMCW) radars and Ultra-Wideband (UWB) radars. Moreover, different carrier frequencies have been used, from 2.4 GHz and 5.8 GHz from the ISM band, to the milimeter wave range. In this work, only solutions based on the CW radars usage are considered.

Despite the countless applications of these systems and the great contribution for the society, there are still emerging challenges that need to be overcome to improve the algorithm robustness and extracted signals accuracy. Bio-signals have low amplitude and the modulation present in the baseband signal is very close to DC. Hence they are highly sensitive to several sources of noise, such as clutter from the scenario reflections, which contributes to the DC level in the baseband signals. The DC component interferes in the signal recover and thus, it should be eliminated.

Furthermore, the presence of Random Motion (RM) in the extracted signal produces a significant source of noise for an accurate detection [9]. This issue limits the range of possible applications of the bio-radar. For example, in overnight applications, it is natural for the patient to roll over in bed, interfering with the acquired signal. There are also cases where the radar operation mode can become a noise source. These cases gather all monitoring environments that are not stationary, for example in rescue missions where the search for survivors is more efficient if a hand-held radar is used or in through-wall military applications. Likewise, vehicular applications, like conventional cars for driver’s monitoring, or in ambulances where a contactless monitoring could be advantageous. Therefore, RM is unavoidable in the most of bio-radar applications and should be considered. The elimination of the interference caused due to RM is a current challenge in this research field.

Several approaches were already explored and they are reviewed and analyzed in this paper. The previous attempts can be divided mainly in three different approach categories: hardware-based solutions, among them the usage of external modules, the implementation of signal processing techniques and the usage of beamforming techniques and other methods based on antenna handling.

As RM we will consider not only the large-scale motions of the body, caused by other body parts rather than the chest-wall or when the subject is walking, but also the motion of the proper radar device during its usage, considering also noisy monitoring environments.

This paper is organized as follows: in Section 2 the mathematical model of bio-radar is presented, with a goal to better understand how the signals behave in these systems. A simulation is done in MATLAB to illustrate the influence of each signal parameter, considering first an ideal scenario and then, the distortion caused by the presence of RM is also depicted. Then, an overview on the previous approaches starts in Section 3, which is focused on solutions with external modules beside the conventional transceiver and antennas. Then, in Section 4 different signal processing algorithms and techniques are analyzed and finally beamforming attempts are reviewed in Section 5. In the end, the conclusion is presented regarding the overall analysis, along with some challenges for future work.

## 2. Signal Model for the Bio-Radar Channel Response

The block diagram presented in Figure 1 represents the operation principal of a bio-radar implemented with a SDR front-end. As mentioned previously, the SDR allows the digital signal processing implementation, so the mathematical model presented in this section distinguishes stages with digital signals and real signals, by using discrete-time domain equations and time-domain equations, respectively.

Bio-radar signal model starts with a baseband signal that is generated digitally. The signal is a complex sinusoid with angular frequency $\omega_{0}$, and it is represented by (1),

(1) $$s\left(n\right)=e^{j\omega_{0}n}.$$

Signal s(n) is then modulated with an In-phase and Quadrature (IQ) modulation, with a carrier frequency $\omega_{c}$, leading to the signal (2), which is transmitted towards to the target.

(2) $$x\left(t\right)=\cos[\left(\omega_{0}+\omega_{c}\right)t]$$

The received signal (3), encompasses the time variant signal $r_{0}\left(t\right)$ correspondent to the chest-wall reflection and the stationary signal $r_{1}\left(t\right)$ which represents the sum of the total sources of clutter.

(3) $$\begin{array}{cc} r(t) & =r_{0}\left(t\right)+r_{1}\left(t\right)\\ & =A_{0}\cos\left[\left(\omega_{0}+\omega_{c}\right)t+\varphi \left(t\right)\right]+A_{1}\cos\left[\left(\omega_{0}+\omega_{c}\right)t+\theta_{1}\right], \end{array}$$

where $A_{0}$ and $A_{1}$ are the amplitudes of the received signal from the subject and clutter, respectively, φ(t) is the phase change function which contains the respiratory information and $\theta_{1}$ is the phase change due to clutter. This phase change term can be expressed as $\theta_{1}=4\pi d_{1}/\lambda$, considering that the clutter source is located at a distance $d_{1}$ from the radar and λ is the wavelength. After its reception, signal r(t) is IQ demodulated resulting in (4), and it is sampled at the same sampling rate $f_{s}$ used in the transmission channel.

(4) $$g\left(n\right)=g_{0}\left(n\right)+g_{1}\left(n\right)=A_{0}e^{j\varphi (n)}+A_{1}e^{j\theta_{1}}$$

The phase change function φ(n), results from the chest-wall motion, which changes the wave traveled distance and hence modulates the reflected signal. Thus, the phase change function can be described by (5):

(5) $$\varphi \left(n\right)=\theta_{0}+\frac{4\pi d(n)}{\lambda },$$

where $\theta_{0}$, is the phase corresponding to the average distance traveled by the wave, expressed as $\theta_{0}\;=\;\left(4\pi d_{o}/\lambda \right)+\phi$, considering the nominal distance between the radar and the target, $d_{o}$, and the phase shift at the target’s surface, ϕ. The respiratory component is described by 4πd(n)/λ. In [14,15], simulations show that the respiratory signal should not be modeled as a simple sinusoid function, so its model should be defined as a half-cycle sinusoid raised to the $p_{th}$ power, as in (6)

(6) $$d\left(n\right)=a_{r}\left(1-\sin^{p}\left(\pi f_{1}n\right)\right),$$

where $a_{r}$ is the amplitude of the chest movement and $f_{1}$ is the breathing rate.

**Figure 2:**
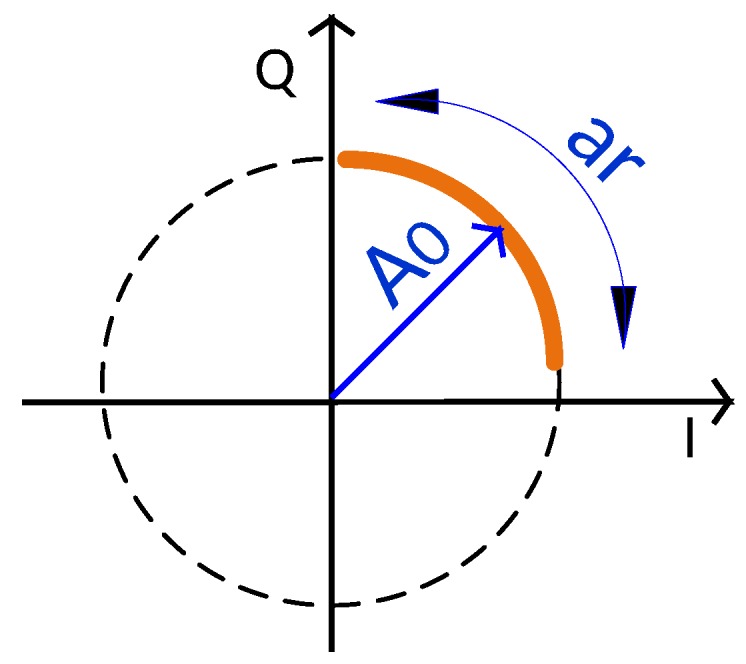
Representation of the phase variation in the complex plane due to the target’s motion, considering a scenario without clutter.

Figure 2, represents the phase variation due to the target’s motion, which consists of an arc in the complex plane. In an ideal scenario (without clutter), the arc fits to a perfect circle centered on the origin. The length of the arc is proportional to the amplitude of the respiratory signal, $a_{r}$, depending on the wavelength of the carrier. Higher carriers lead to higher sensitivity to detect weak motions, which means that regarding the same motion amplitude ($a_{r}$), shorter wavelengths create wider arcs rather than longer wavelengths. The radius of the arc is the received signal’s amplitude $A_{0}$. The arc position in circle varies with the distance between the radar and the target, defined as $d_{0}$.

In real-world scenarios there are some effects that change the obtained arc and could influence in the accuracy of the breath rate extraction. For instance, the IQ imbalance effect occurs when both real and imaginary parts do not have the same amplitude and the phase relationship is not exactly $90^{\circ }$. Hence, the formed arc fits an ellipse instead of a circle.

Besides the IQ imbalance, the scatter signals from stationary objects within the radar range (also known as clutter) will be perceived as a DC component in the baseband signal spectrum, which inhibits the proper signal extraction.

In the subsequent sections, simulations of the mathematical model will be presented, similarly to the ones presented in [12], but considering now the respiratory signal model given by Equation (6). These simulations aim to better understand the effect of each parameter in the acquired signal, as well as the distortions caused by the RM occurrence. The DC component and IQ imbalance problems are not focused on in this work.

### 2.1. Simulation of the Mathematical Model Considering an Ideal Scenario

First of all, an ideal channel is considered and the parameters of the baseband signal will be studied. For this purpose, a MATLAB simulation was performed. Once the DC component is not going to be explored in this work, the $g_{1}\left(n\right)$ term from (4) is neglected, so the baseband signal can be re-written as (7), using (4)–(6):

(7) $$g\left(n\right)=A_{0}e^{j(\frac{4\pi d_{o}}{\lambda }+\phi +\frac{4\pi d(n)}{\lambda })}$$

**Figure 3:**
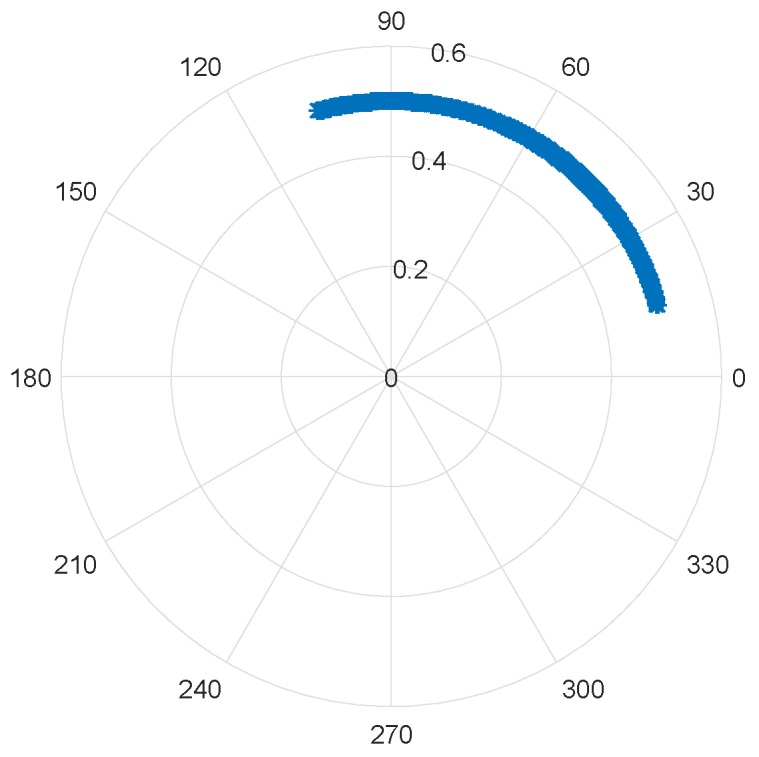
Simulation of the phase variation in the complex plane due to the target’s motion.

In order to understand the influence of each parameter, the MATLAB simulation started with the following parameters:

- Sampling frequency—$f_{s}=1000$ Hz;
- Wavelength—λ=0.0517 m (for a carrier frequency equal to $f_{c}=5.8$ GHz);
- Received signal’s amplitude—$A_{0}=0.5$;
- Distance between the target and the radar—$d_{o}=3$ m;
- Chest-wall motion’s amplitude—$a_{r}=\lambda /4$ m;
- Initial phase shift—ϕ=π/12 rad;
- Respiratory frequency—$f_{1}=0.4$ Hz;
- $p_{th}$ power of the half-cycle sinusoid—p=4;

Similarly to Figure 2, the phase variation of this baseband signal is depicted by the polar plot in Figure 3.

**Figure 4:**
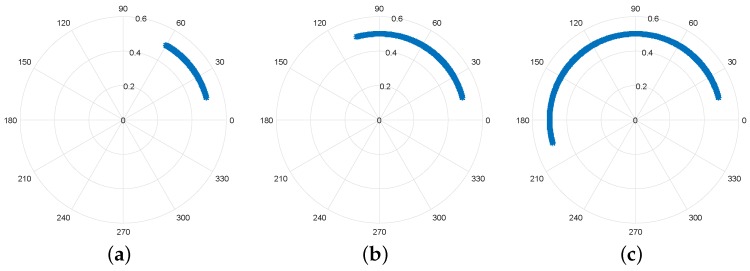
Variation of the arc length with chest-wall motion amplitude: (**a**) $a_{r}=\lambda /8$ m; (**b**) $a_{r}=\lambda /4$ m; (**c**) $a_{r}=\lambda /2$ m.

As mentioned previously, the arc’s length is proportional to the chest-wall motion amplitude, $a_{r}$, which has also a direct relation with the wavelength. In other words, if $a_{r}$ reaches the wavelength value, a full circle is perceived in the complex plot rather then an arc. Figure 4 illustrates the relation between the arc’s length and the $a_{r}$ value.

**Figure 5:**
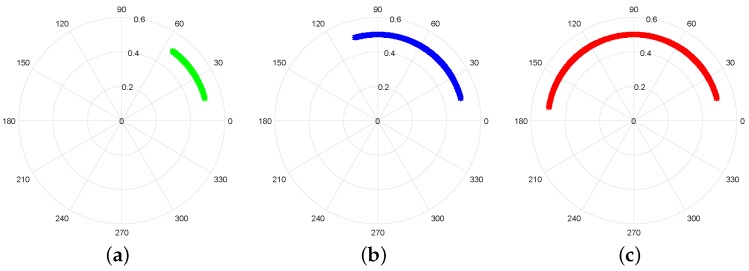
Variation of the arc length with the wavelength for the same $a_{r}$: (**a**) $f_{c}=2.5$ GHz; (**b**) $f_{c}=5.8$ GHz; (**c**) $f_{c}=10$ GHz.

If the same value of $a_{r}=\lambda /4$ m is kept constant, it is also possible to understand how different wavelengths can change the sensitivity to detect weaker motions, as shown in Figure 5. For example, a carrier frequency equal to $f_{c}=10$ GHz provides longer arc length, when compared with the $f_{c}=2.5$ GHz case, considering the same amplitude $a_{r}$.

**Figure 6:**
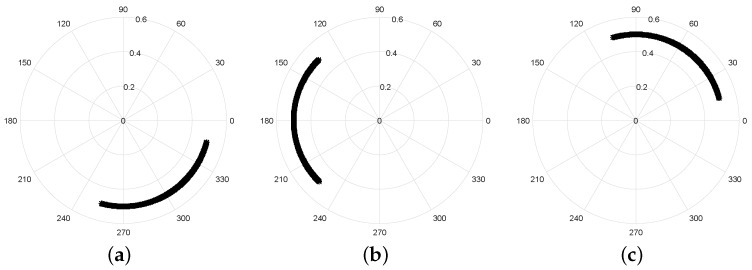
Variation of the arc position of the nominal distance $d_{0}$: (**a**) $d_{0}=1$ m; (**b**) $d_{0}=2$ m; (**c**) $d_{0}=3$ m.

The nominal position of the target relative to the radar, $d_{0}$, changes the position of the arc with respect to the full circle. This effect can be observed in Figure 6, but the information is the same.

**Figure 7:**
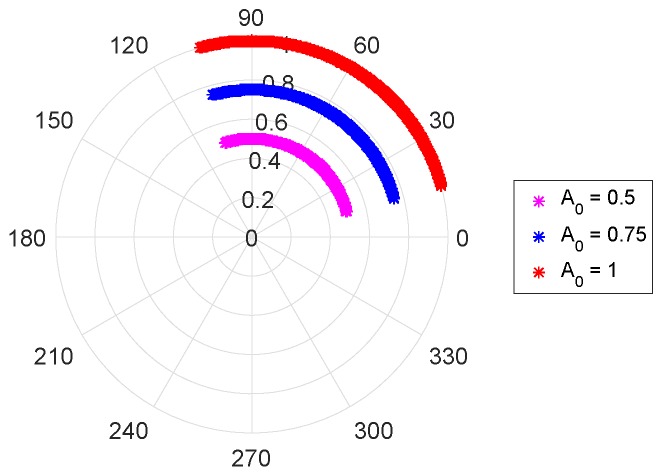
Variation arc radius with the received signal amplitude $A_{0}$.

Finally, the amplitude $A_{0}$ corresponds to the radius of the arc, as depicted in Figure 7.

### 2.2. Random Motion Signal Model

**Figure 8:**
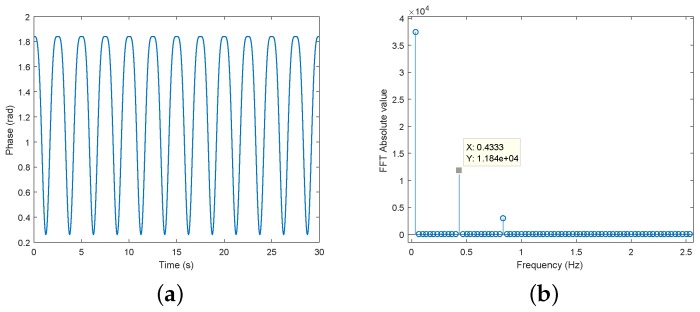
Respiratory signal recover: (**a**) Respiratory waveform; (**b**) its spectrum (zoomed in) with the respiratory component $f_{1}=0.4333\;\mathrm{Hz}$ represented.

In this subsection, a simulation of the mathematical model is performed considering now the impact of the RM occurrence in the acquired signal. For comparison purposes, Figure 8a shows the expected respiratory signal after its recover, considering an ideal scenario, and its spectrum is represented in Figure 8b. The phase demodulation method considered was the arctangent demodulation, first proposed in [16], which is the most commonly used.

As we have seen so far, during the monitoring period, it is natural that the patient does not stay completely still. For example, in sleeping monitoring, patients roll over in bed mostly due to comfort reasons. These motions appear randomly and have much higher amplitude and frequency than bio-signals. Thus, the mathematical model of the total signal can be seen as a concatenation of two different signals: the respiratory signal represented by Equation (6), where the values of $a_{r}$ and $f_{1}$ are the same used in the previous simulation, and the RM signal $d_{2}\left(n\right)$, (8), which was modulated as a high frequency sinusoid, with an amplitude proportional to the wavelength (the same considered in the previous simulation $f_{c}=5.8$ GHz).

(8) $$d_{2}\left(n\right)=a_{r2}\cos\left(2\pi f_{2}n\right)$$

**Figure 9:**
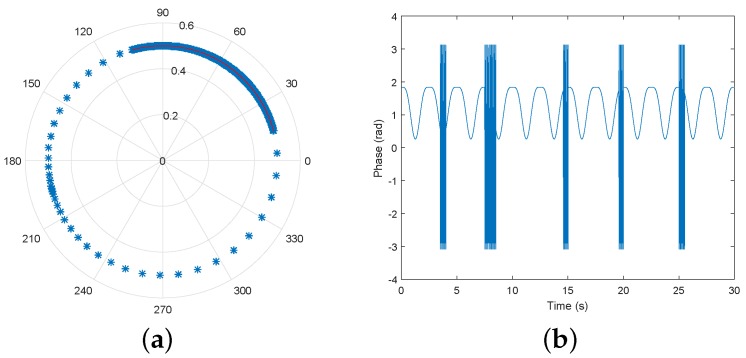
Respiratory signal distorted by RM: (**a**) Effect on the polar plot; (**b**) Waveform after phase demodulation.

For simulation purposes, the RM frequency $f_{2}=10$ Hz was considered, and the acquired signal, under these circumstances can be visualized in Figure 9.

It is possible to observe that the RM have much higher amplitude, when compared with the respiratory signal, hence a full circle is described in the complex plane, and the original arc is also perceived.

**Figure 10:**
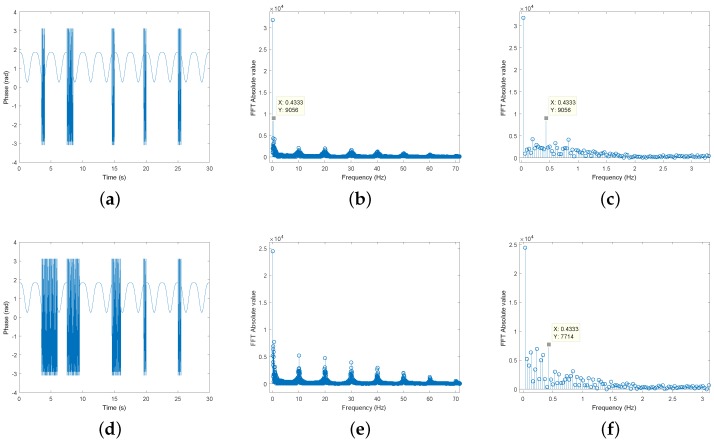
Respiratory signal distorted by RM: (**a**) RM signal waveform with a soft level of noise; (**b**) Spectrum with a soft level of RM noise; (**c**) Zoomed in spectrum for a soft level of noise; (**d**) RM signal waveform with a higher level of noise; (**e**) Spectrum with a higher level of RM noise; (**f**) Zoomed in spectrum for a higher level of noise

Figure 10 shows the impact of the RM component in the signals spectrum. First, the spectrum from Figure 9 can be observed in Figure 10b and its zoomed version in Figure 10c. In this case, it is possible to observe the harmonics of the RM signal over the spectrum and there are more spectral components, when compared with the spectrum from Figure 8b. Moreover, it is still possible to distinguish easily the respiratory component, marked as f=0.4333 Hz.

However, if the RM signal is increased as shown in Figure 10d, also the spectrum will be affected. In this case, the harmonics content will increase and other spectral components can superimpose with the desired signal, as represented in Figure 10f. Thus, it is possible to conclude that more periods of motion can lead to massive signals distortion and prejudice in the vital signal rate computation. In this sense, it is crucial to find solutions to compensate the RM distortion in real time applications, which aim to enhance the accuracy of these systems.

## 3. Hardware Based Solutions for the Motion Compensation

Regarding the solutions so far developed and proposed to overcome the RM detection and mitigation, they have been implemented with different strategies. In this section, applications using hardware modifications and including external modules are going to be reviewed.

In [17] it is shown that RM can be distinguished from the chest-wall displacement, by identifying other motion patterns. For this purpose two radars were used, located in the front and in the back of the patient. The signals received by both receivers due to the physiological functions are in phase, differing only in the sign, once the back and chest move in opposite directions. Thus, if the subject moves randomly, an out of phase signal will be received and a RM case will be detected. The motion effect will cause a frequency shift in the baseband spectrum, which can also be canceled by doing the convolution of both acquired signals.

It is important to notice that once two radars are used and they are faced to each other, the polarization of their antennas have to be orthogonal in order to prevent interference. Also the wavelength used by both transceivers should be approximately the same.

The work presented in [17] is also focused on an important detail, which is the phase demodulation technique. A commonly used technique is the arctangent demodulation (AT) [16], however they point out that with this method, the signal is strongly affected by the DC offset and a prior calibration is required. Therefore, they also present other demodulation technique called Complex Signal Demodulation (CSD), which combines both IQ parts of the complex signal and computes directly the Fourier transformation taking into account the Bessel function. This method is not susceptible to the DC component presence. Considering the advantages and disadvantages of the AT and CSD method, the applicability of them in situations where RM occurs is studied in [18], using the same set-up used in [17]. They verified that if the DC is correctly removed, it is possible to recover vital signals without interference. However, it is strongly affected if the DC is present.

Also in [19], two radars located in the same positions as in [17] are being used, but in this case a radar with FMCW operation mode is employed. They do a range-bin alignment to combine the range histories. This system estimates the target distance variation seen from both radars and in this way mitigate RM distortion to recover the vital signals accurately.

A different hardware architecture was proposed in [20]. It was composed by a Self Injection-Locked (SIL) radar with a single antenna for both transmission and reception. Their set-up approach for vital signals acquisition, was to place two radars with this configuration, faced to the front and back of the patient and thus acquire RM reflections from two different angles. In this way was possible to cancel the RM component and restore the useful vital signals. Although this set-up configuration has some limitations regarding practical applications, such as for bedridden patients. In this sense, a different antenna configuration was first proposed in [21] and improved in [22]. The antennas were side by side, rather than being located in front and back of the patient, and had different gain features. Thus, vital signals and RM have different weights and the vital signal elimination is avoided. This self-signal mitigation could happen if the antennas had the same features, because there are two SIL paths that are out of phase, with the aim to cancel RM signal.

Nonetheless, these set-ups based on SIL hardware architectures that have been presented so far, were too sensible to large scale motions. Therefore, despite its limitations in practical implementation, the configuration of front-back antennas was restored in [23]. The conducted procedure was the following: a CW signal is transmitted towards the chest front and the received echo is retransmitted toward to the back of the patient. The second reflected signal is injected to a injection-locked oscillator and once this signal contains two Doppler phase modulations (from front and back motions, respectively) they can be combined to either cancel in case of RM occurrence or to sum themselves once with this configuration vital signals that have the same phase in both illuminated sides.

In [24] a solution based on dual helical antennas is proposed, to illuminate the chest-wall in two different locations and thus implement a differential measurement, which is only focused on in the cardiac signal. One of the antennas is directed to the heart and the beam of the second antenna is focused more below. In this way is possible to cancel background noise or any other motion that is perceived equally by both antennas. Two separated radars are used, operating in slightly different frequencies (2.45 and 2.5 GHz, respectively). The helical antennas are cross-polarized in order to avoid self-interference. The differential subtraction aims to recover the heartbeat signal without the interference caused by other motion. Although if the random motion is not perceived equally in the both radars, these signals can not be entirely subtracted. In this sense, a failure detected by the authors, was the difficulty to cancel the breathing signal from the cardiac one, since the motion from the diaphragm is different from the entire chest-wall motion.

In [16,17,18,19,20,21,22,23,24] it was proved that information provided by two radars is enough to help in the RM mitigation. Although RM is unpredictable and can actually occur in multiple directions. As so, in [25] four radars are used and the combination of the four acquired signals can cancel the noise that results from the RM. Since the radars are placed as directional pairs, i.e. in the front-back direction and in the right-left direction respectively, the motion component in the acquired signal has opposite signs on each pair. Hence, the four signals are in phase and the RM signal is out of phase. With this phase relation, the multiplication of the four complex signals will cancel the RM distortion.

Other solutions to distinguish vital signs from RM were proposed, using auxiliary hardware beside extra radars. For example, in [26] harmonic tags placed on the subject’s chest were used. The tag is faced to the receiver and it converts the incoming signal in harmonics. Then, the second harmonic is transmitted back to the receiver. The tag only detects the chest motion due physiological functions. In this case only the respiratory function is considered, hence other body motions or even moving objects present in the range of the radar are not detected.

The solution presented in [27] intends to overcome the motion effect that occurs due to specific radar operational conditions. For example, when these systems are used as a hand-held device in rescue operations, where the user hand-shake can cause disturbance. This set-up consists in the conventional bio-radar system with one transceiver transmitting a CW signal towards the target and an auxiliary sensor node located next to the target, which aims to attenuate the RM effect. It receives the signal transmitted by the transceiver and the signal reflected by the target, which is phase modulated by the chest-wall motion simultaneously. If the transceiver moves randomly, both signals in the sensor node will be affected by the distance change and hence higher phase modulation will corrupt the bio-signal. The signal is down-converted in the sensor node by mixing it with itself. If the distance between the transceiver and the target is extremely large and if the sensor node is really close to the target, it can be assumed that both target and sensor are at the same distance in relation to the transceiver. Thus, the received signal depends only on the distance between the target and the sensor node and hence is not affected by the transceiver motion.

The antenna’s motion was also considered as source of disturbance in [28]. In this work, an accelerometer chip attached to the transceiver antenna records the vibration during the monitoring process. The information is processed later in order to estimate the total distance variation, caused by the physiological motion and by the antenna’s motion separately improving the vital signals information extraction.

In [29,30], a camera is used to measure the human body motion and by using a simple motion extraction algorithm, the phase information needed for phase compensation is fed back to the radar and used later for the RM cancellation algorithms. The authors explored three different strategies to compensate the RM effect. The first approach compensates for the phase directly in the RF front-end, by using a phase shifter and in this way the circuit saturation that can occur due to RM is avoided. The second alternative is the phase compensation in the baseband output signal, if there is no saturation in the front-end. Finally, the third alternative is the motion signal cancellation in the demodulated signal. The latter approach would be the most straightforward solution, although since it is implemented after the arctangent demodulation, the large movements will cause wraps in the demodulated signal, which can still be corrected using unwrapping or DACM algorithms [31].

The camera used to evaluate the human body motion was a common Cannon camera in [29] and in [30] the possibility to extend these technologies to smartphones applications was exploited, by using an iPhone 5 camera. In practice, the phase information can be retrieved from the variation of the number of pixels occupied by the subject in the video that changes with his/her motion.

Solutions that gather external hardware components beside the transceiver have been seen so far. Despite the good results in [16,17,18,19,20,21,22,23,24,25,26,27,28,29,30] and the effectiveness in the random motion compensation, the requirement of external modules turns the implementation too complex. In [16,17,18,19,20,21,22,23,24,25] is required the synchronization of all acquisitions which is difficult to achieve. In [26,27] it is necessary to either have contact with the patient or to predict his/her location, so the concept of non-contact and remote monitoring is not totally applicable. Moreover, the work in [27] solves one source of motion, but not body motion as well.

## 4. Digital Signal Processing Methods for Motion Mitigation

In the following works, different solutions are proposed using only software processing, which simplify the implementation regarding the hardware.

In [32] the usage of different carrier frequencies was exploited and the different system behavior due to the skin penetration was evaluated. A two-frequency radar was proposed, operating at $f_{1}=800$ MHz and $f_{2}=2.4$ GHz, respectively. Longer wavelengths are capable to penetrate the skin, so $f_{1}$ is used to collect the reflections that occur in the heart. On the other hand, since the lungs are closer to the surface, lower wavelengths are enough and $f_{2}$ is used for this purpose. The system developed in this work, transmits two signals toward the subject and receives the reflected signals mixed. The result only gathers the difference between the distance to heart and to lungs, which do not depend on the distance between the radar and the subject, so the change in the traveling distance of the transmitted waves due to RM will not be perceived in the recovered signal.

The solution proposed in [33] can compute the respiratory rate even if there is interference due to RM. In this work, only 1-D body motion is considered, i.e. the motion relative to the target forward and backward motion. It was concluded that this type of motion shifts the position of the spectral information. The CSD method [17], was used to extract the phase information. The spectral peaks centered in DC, which correspond to the respiratory and heartbeat component respectively, are shifted to the positive axis if the target moves towards the radar and to the negative axis if the target moves backwards. Although the distance between peaks remains the same. Thus, the respiratory rate can be obtained by the computation of the mean value of the two respiratory peaks, even if RM happens.

In [34] a curve fitting approach is used to compensate large motions of the patient. They have considered that large scale motions affects the tendency of the vital signals, or in other words, its instantaneous mean value in time. Their approach consisted of the division of the acquired signals in sub-wavelength signals and after the DC removal and phase demodulation, a polynomial fitting was performed on each segment of data to find that tendency, which is then subtracted from the raw data to retrieve the useful information.

In [35] a cyclostationary approach is implemented in order to retrieve useful information from bio-signals without any distortion treatment, even in noisy monitoring scenarios or in case of RM. The cyclostationary process uses the cyclic statistics to analyse signals with hidden periodicities. The heartbeat and respiratory signal are waveforms that are subjected to some variations, although it is possible to perceive a periodic variation of some waveform parameters, if the observation window is selected with care. This allows the application of these methods in these vital signals. In order to see the effect of motion in the spectral computation, both Spectral Correlation Function (SCF) and Fast-Fourier Transform (FFT) were obtained under low Signal-to-Noise Ratio (SNR) and low Signal-to-Interference-plus-Noise Ratio, (SINR). Results showed that in case of RM, the spectral component of bio-signals is not apparent using only the FFT analysis, but is noticeable in the SCF plot. Thus the SCF of a cyclostationary signal is insensitive to all noncyclic components, such as RM.

In [36], the heartbeat signal is recovered using the Empirical Mode Decomposition (EMD). This method decomposes the signal into narrow band components, also known as Intrinsic Mode Functions (IMF) [37]. The signal at the input of the system is decomposed in several IMFs and then the motion artifact can be located. Usually the RM is present in the first IMF, (verified by visual inspection of this method simulation in MATLAB), hence the sum of the remain IMF reconstructs the bio-signal without the external motion interference.

In [38] the DC cancellation problem under RM is solved. In order to save computational resources, the algorithm proposed by this work is applied within two stages. The first stage divides the original dataset in multiple data segments, by time windowing. The window length was selected considering the average respiratory frequency –0.25 Hz. Then the mean value of the original data is subtracted from each data segment. If there is any abrupt change in the respiratory signal caused by the RM, this first stage is not enough, therefore additional processing is required. The relative strength is evaluated, to find the segments that contain RM. The interval of samples that delimits the motion period is identified for each motion segment and thereafter a sub-segment is defined considering these boundary samples. The mean value is subtracted to each sub-segment and the signal is reconstructed by the concatenation of all segments and sub-segments.

The feasibility of the bio-radar usage to monitor sleeping rodents is evaluated in [39], after the administration of medicines that aims to treat in the future patients with sleeping disorders. During the monitoring period, it is natural that the rodent moves, causing sudden peaks in the acquired signal. The intention of the proposed method is to reject automatically the resulting distortions and restore the useful signal. This method is based on delayed-windowing. A window is applied in the signal with a length equal to *M* and moves across the signal samples, generating a matrix with a Toeplitz structure. Then, the energy of each column is computed and a threshold is defined by its mean value. The samples of the output signal are forced to zero, everytime it reaches that threshold value.

In addition to sleeping monitoring applications [40] developed a prototype to detect the respiration and other locomotor activity in sleeping patients crew members from a spaceship. The authors evaluated that when a random motion occurs, the correspondent spectral component is around 1 Hz, while during the normal breathing period it do not exceed 0.6 Hz. Therefore, an algorithm based on these spectral differences is proposed. A high-pass filter is applied to the signal and its filtered output is subtracted from the original signal. This procedure will isolate the artifact component, which can be removed afterwards.

An approach that is completely different from every other that we have seen so far, is presented in [41] and uses a bootstrap-based Generalized Warblet Transform (GWT) method, to obtain a time-frequency representation of the acquired signal. GWT have revealed to be an effective tool to deal with oscillating time-frequency patterns of periodic signals [42]. Moreover, bootstrap-based algorithms can be used to enhance the GWT estimation, once it consists in a statistical resampling method and a useful tool when is not possible to make measurements in real-time. The authors have modeled two vital-signals, as two different oscillating components, and the RM as a high value of SINR. It was possible to extract vital signals with a reduced error, even with high values of SNR and SINR. The prototype proposed in [41] uses a heterodyne configuration, rather than the homodyne which was the most used in the works that we have seen so far. This was the chosen option to help in the mitigation of the quadrature imbalance and DC offsets occurrence.

The RM mitigation using signal processing methods revealed promising solutions, although some drawbacks should be pointed out. It is still difficult to separate the heartbeat signal from the respiratory signal and the RM occurrence does not enhance this task. Methods like [32,36] focus only in the heartbeat signal, which has a low SNR due to the higher reflection on lungs. Therefore, the SNR should be increased. In the EMD method, the presence of the breathing component also difficult the IMF decomposition, so it was needed to apply a high pass filter in the signal to remove this component. The EMD method was limited regarding the RM amplitude, because high amplitude motions require higher frequency separation to isolate each spectral components.

The hardware imperfection can inhibit the optimal performance of theses methods, as well. For example, in [33] the interference of IQ imbalance originates peaks in the opposite axis and this effect can corrupt the rate computation.

Moreover, some algorithms and techniques could be heavy computationally, such as in [34].

## 5. Phased-Array Antennas, Beamforming Techniques and Other Antenna Handling Methods

In [43,44], prototypes that use beam-steerable phased array antennas are presented. With these approach it is possible to scan different detection angles and thus, determine which one is the best angle of detection. Firstly, a prototype was developed in [43] and a performance comparison is made between the usage of phased-array antennas and fixed beam antenna. Experimental tests were conducted inside an anechoic chamber and the results showed more accuracy in the respiratory signal acquisition when the antenna beam is directed to the best detection angle. Then in [44], an improved version of the prototype presented in [43] was used to implement an automatic beam steering system, focused on sleeping applications where the naturally motion of the patient is considered. Tests were performed in a non-laboratory environment, more specifically in a large office cubicle to simulate a bedroom and volunteers were laid down. Here the performance of a fixed-beam system was also compared with a automatic beam steering system, where the later stands out with optimal results. The implemented algorithm selects the best angle of detection after evaluating 7 different angles using standard deviation and median, and vital signals are then recorded during 60 s using the selected angle. In this work the author was only focused on in the cardiac signal acquisition.

Also in the context of long term monitoring applications, such as overnight sleeping monitoring, adaptive solutions using phased-array techniques have been explored in [45]. Based on the prototype presented in [44], a single PCB was developed with a view to decrease the front-end size, minimize cables loss and improve signals detection accuracy. Moreover, it is proposed an intelligent automatic beam-steering Doppler sensor that will perform a 7-angle sweep everytime it detects a large movement or a significant reduction in the signal strength. After the sweep, it computes the standard deviation or the variance ratio of the received signal and the angle with the highest value is chosen to steer the beam in that direction. The motion detection runs in parallel with the bio-signals acquisition and processing. Although each re-sweep time was measured and it takes 350 s to cover all the 7 angles, which is an extremely long-time consuming.

Regarding other techniques with antenna handling, the work presented in [46] was more focused on the proper separation of vital signals from unwanted motion of other body parts, instead of considering other RM sources. A Single-Input Multiple-Output (SIMO) system was used along with the Blind Source Separation (BSS) implemented on the incoming reflections from multiple body parts, in order to enable the separation of the chest-wall motion from the hand shake. The set-up implemented had a single transmitter and two receivers and the selected BSS algorithm was the Real Analytical Constant Modulus Algorithm (RACMA), which is able to separate two sources of signal by adjusting the beam direction adaptively, according to the phase and amplitude of each source. It is important to note that this implementation was limited in area resolution to separate various motion sources from the same body, which means that the hand had to be separated from the body, otherwise its motion would not be detected as a separate motion.

In general, phased-array antennas, beamforming techniques and other forms of antenna handling are advantageous under the circumstances depicted in this article. Regarding the antenna dynamics, fixed beam antennas performance was compared with a beam-steerable phased array antennas, which proved to have more accurate results. Experimental tests were conducted using multi-antenna systems and it was possible to separate RM from different body parts. Although high resolution is required to distinguish different body parts that are closed to each other. Moreover, more simulations are required in order to characterize different RM sources, once the solutions that we have seen so far were more focused on the subject RM and it lacks solutions that also encompasses noisy monitoring scenarios.

## 6. Conclusions

Non-contact system for vital signals measurements such as the so called Bio-Radar, is a promising tool for the enhancement of healthcare systems and can be integrated in other systems to improve people life style, as well. Hence, it can be seen as a research field that have been deeply studied by many researchers.

The distortion caused by RM, is one inherent problem that does not have yet a straightforward solution. In this article, a review focused on the implemented methods for the motion detection and compensation is done. First solutions based on hardware implementations were presented. This solutions could bring implementation complexity and hardware imperfections are easily perceived in the acquired signal. Moreover, prototypes with multiple antennas surrounding the subject were proposed, a long with the usage of other auxiliary equipment such as tags or cameras, but these solutions require complex and expensive implementation. Then, several signal processing methods were presented that only require a single radar and hence are simpler to implement, but can have high computational cost. Nonetheless, these methods have the capacity to solve several types of RM sources and could be more effective in the general enhancement of bio-radar systems and their robustness. Beam-forming techniques were also considered and revealed being more suitable for long-term monitoring applications and successful in the compensation of random body motions.

Even though, several solutions have been proposed so far, there is not yet a straightforward solution that is efficient to all types of RM sources and that turn the system more suitable for all types of applications. Thus as future work, it is expected to find a solution that gathers the contributions of each solution and could encompass a compensation for all the different sources of RM. The most promising solution should combine beamforming techniques to follow the subject in case of body RM, multiple antennas handle to take advantage of the constructive multipath and a adaptive filtering system, performed by means of digital signal processing algorithms to compensate RM caused due to the noise conditions of operation.

## References

[1] Boric-Lubecke O., Lubecke V., Droitcour A., Park B., Singh A. (2015). Doppler Radar Physiological Sensing.

[2] Malafaia D., Oliveira B., Ferreira P., Varum T., Vieira J., Tomé A. (2016). Cognitive bio-radar: The natural evolution of bio-signals measurement. J. Med. Syst..

[3] Gouveia C., Malafaia D., Vieira J., Pinho P. (2018). Bio-Radar performance evaluation for different antenna design. URSI Radio Sci. Bull..

[4] Lin J.C. (1975). Non-invasive microwave measurement of respiration. Proc. IEEE.

[5] Lin J.C., Dawe E., Majcherek J. (1977). A non-invasive microwave apnea detector. Proceedings of the San Diego Biomedical Symposium.

[6] Lin J.C., Kiernicki J., Kiernicki M., Wollschlaeger P.B. (1979). Microwave apexcardiography. IEEE Trans. Microw. Theory Tech..

[7] Chan K.H., Lin J.C. (1987). Microprocessor-based cardiopulmonary rate monitor. Med. Biol. Eng. Comput..

[8] Li C., Yu X., Lee C.M., Li D., Ran L., Lin J. (2010). High sensitivity software configurable 5.8 GHz radar sensor receiver chip in 0.13 *μ*m CMOS for non-contact vital sign detection. IEEE Trans. Microw. Theory Tech..

[9] Li C., Lubecke V., Boric-Lubecke O., Lin J. (2013). A review on recent advances in doppler radar sensors for noncontact healthcare monitoring. IEEE Trans. Microw. Theory Tech..

[10] Malafaia D., Vieira J., Tomé A. (2015). Improving performance of bio-radars for remote heartbeat and breathing detection by using cyclostationary features. Proceedings of the International Joint Conference on Biomedical Engineering Systems and Technologies.

[11] Droitcour A., Boric-Lubecke O., Lubecke V., Lin J., Kovacs G. (2004). Range correlation and I/Q performance benefits in single-chip silicon doppler radars for non-contact cardiopulmonary monitoring. IEEE Trans. Microw. Theory Tech..

[12] Gouveia C. (2017). Bio-Radar. Master’s Thesis.

[13] Gouveia C., Malafaia D., Vieira J., Pinho P. Influence of radiation pattern in the performance of bio-radar. Proceedings of the IEEE International Symposium on Antennas and Propagation.

[14] Morgan D.R., Zierdt M.G. (2009). Novel signal processing techniques for doppler radar cardiopulmonary sensing. Signal Process..

[15] Zakrzewski M., Raittinen H., Vanhala J. (2012). Comparison of center estimation algorithms for heart and respiration monitoring with microwave doppler radar. IEEE Sens. J..

[16] Park B., Boric-Lubeck O., Lubecke V. (2007). Arctangent demodulation with DC offset compensation in quadrature doppler radar receiver systems. IEEE Trans. Microw. Theory Tech..

[17] Li C., Lin J. Complex signal demodulation and random body movement cancellation techniques for non-contact vital sign detection. Proceedings of the IEEE MTT-S International Microwave Symposium Digest.

[18] Li C., Lin J. (2008). Random body movement cancellation in Doppler radar vital sign detection. IEEE Trans. Microw. Theory Tech..

[19] Munoz-Ferreras J., Peng Z., Gomez-Garcia R., Li C. Random body movement mitigation for FMCW-radar-based vital-sign monitoring. Proceedings of the IEEE Topical Conference on Biomedical Wireless Technologies, Networks, and Sensing Systems (BioWireleSS).

[20] Wang F.-K., Horng T.-S., Peng K.-C., Jau J.-K., Li J.-Y., Chen C.-C. (2011). Single-antenna Doppler radars using self and mutual injection locking for vital sign detection with random body movement cancellation. IEEE Trans. Microw. Theory Tech..

[21] Tang M.-C., Kuo C.-Y., Wun D.-C., Wang F.-K., Horng T.-S. Same side dual SIL-radar system for real-time vital sign monitoring with random body movement cancellation. Proceedings of the IEEE MTT-S International Microwave Symposium (IMS).

[22] Tang M.-C., Kuo C.-Y., Wun D.-C., Wang F.-K., Horng T.-S. (2016). A self-and mutually injection-locked radar system for monitoring vital signs in real time with random body movement cancellation. IEEE Trans. Microw. Theory Tech..

[23] Tang M.-C., Wang F.-K., Horng T.-S. (2017). Single Self-Injection-Locked Radar with Two Antennas for Monitoring Vital Signs with Large Body Movement Cancellation. IEEE Trans. Microw. Theory Tech..

[24] Fletcher R., Han J. Low-cost differential front-end for Doppler radar vital sign monitoring. Proceedings of the IEEE MTT-S International Microwave Symposium Digest.

[25] Yu X., Li C., Lin J. Two-dimensional noncontact vital sign detection using doppler radar array approach. Proceedings of the IEEE MTT-S International Microwave Symposium Digest (MTT).

[26] Singh A., Lubecke V.M. (2012). Respiratory monitoring and clutter rejection using a CW doppler radar with passive RF tags. IEEE Sens. J..

[27] Mostafanezhad I., Park B., Boric-Lubecke O., Lubecke V.M., Host-Madsen A. Sensor nodes for doppler radar measurements of life signs. Proceedings of the 2007 IEEE/MTT-S International Microwave Symposium.

[28] Mostafanezhad I., Boric-Lubecke O., Lubecke V., Host-Madsen A. Cancellation of unwanted motion in a handheld doppler radar used for non-contact life sign monitoring. Proceedings of the IEEE MTT-S International Microwave Symposium Digest.

[29] Gu C., Wang G., Inoue T., Li C. Doppler radar vital sign detection with random body movement cancellation based on adaptive phase compensation. Proceedings of the IEEE MTT-S International Microwave Symposium Digest (IMS).

[30] Gu C., Wang G., Li Y., Inoue T., Li C. (2013). A hybrid radar-camera sensing system with phase compensation for random body movement cancellation in Doppler vital sign detection. IEEE Trans. Microw. Theory Tech..

[31] Wang J., Wang X., Chen L., Huangfu J., Li C., Ran L. (2014). Noncontact distance and amplitude-independent vibration measurement based on an extended DACM algorithm. IEEE Trans. Instrum. Meas..

[32] Oum J.H., Kim D., Hong S. Two frequency radar sensor for non-contact vital signal monitor. Proceedings of the IEEE MTT-S International Microwave Symposium Digest.

[33] Tu J., Hwang T., Lin J. (2016). Respiration rate measurement under 1-D body motion using single continuous-wave doppler radar vital sign detection system. IEEE Trans. Microw. Theory Tech..

[34] Lv Q., Dong Y., Sun Y., Li C., Ran L. Detection of bio-signals from body movement based on high-dynamic-range Doppler radar sensor. Proceedings of the IEEE MTT-S 2015 International Microwave Workshop Series on RF and Wireless Technologies for Biomedical and Healthcare Applications (IMWS-BIO).

[35] Kazemi S., Ghorbani A., Amindavar H., Li C. (2014). Cyclostationary approach to doppler radar heart and respiration rates monitoring with body motion cancellation using radar doppler system. Biomed. Signal Process. Control.

[36] Mostafanezhad I., Yavari E., Boric-Lubecke O., Lubecke V., Mandiac D.P. (2013). Cancellation of unwanted doppler radar sensor motion using empirical mode decomposition. IEEE Sens. J..

[37] Huang N.E., Shen Z., Long S.R., Wu M.C., Shih H.H., Zheng Q., Yen N., Tung C.C., Liu H.H. (1998). The empirical mode decomposition and the hilbert spectrum for nonlinear and non-stationary time series analysis. Proc. R. Soc. Lond. A Math. Phys. Eng. Sci..

[38] Li Y., Wang G., Gu C., Li C. Movement-immune respiration monitoring using automatic DC-correction algorithm for CW doppler radar system. Proceedings of the IEEE Topical Conference on Biomedical Wireless Technologies, Networks, and Sensing Systems (BioWireleSS).

[39] Anishchenko L., Gennarelli G., Tataraidze A., Gaysina E., Soldovieri F., Ivashov S. (2015). Evaluation of rodents’ respiratory activity using a bioradar. IET Radar Sonar Navig..

[40] Anishchenko L.N., Demendeev A.A., Ivashov S.I. (2013). Use of Radiolocation for Non-contact Estimation of Patterns of Respiration and Motion Activity in Sleeping Humans. Biomed. Eng..

[41] Kazemi S., Ghorbani A., Amindavar H., Morgan D.R. (2016). Vital-sign extraction using bootstrap-based generalized warblet transform in heart and respiration monitoring radar system. IEEE Trans. Instrum. Meas..

[42] Yang Y., Peng Z.K., Meng G., Zhang W.M. (2012). Characterize highly oscillating frequency modulation using generalized Warblet transform. Mech. Syst. Signal Process..

[43] Boothby A., Das V., Lopez L., Tsay J., Nguyen T., Banister R.E., Lie D.Y.C. Accurate and Continuous Non-Contact Vital Signs Monitoring using Phased Array Antennas in a Clutter-Free Anechoic Chamber. Proceedings of the 35th Annual International Conference of the IEEE Engineering in Medicine and Biology Society (EMBC).

[44] Hall T., Nukala B.T., Stout C., Brewer N., Tsay J., Lopez J., Banister R.E., Nguyen T., Lie D.Y.C. A Phased Array Non-Contact Vital Signs Sensor with Automatic Beam Steering. Proceedings of the IEEE MTT-S International Microwave Symposium.

[45] Batchu S., Narasimhachar H., Mayeda J.C., Hall T., Lopez J., Nguyen T., Banister R.E., Lie D.Y.C. Overnight non-contact continuous vital signs monitoring using an intelligent automatic beam-steering doppler sensor at 2.4 GHz. Proceedings of the 39th Annual International Conference of the IEEE Engineering in Medicine and Biology Society (EMBC).

[46] Vergara A., Petrochilos N., Boric-Lubecke O., Host-Madsen A., Lubecke V. Blind source separation of human body motion using direct conversion doppler radar. Proceedings of the IEEE MTT-S International Microwave Symposium Digest.

